# Comparing the circulating immune profile of women with and without recurrent implantation failure: a systematic review and meta-analysis

**DOI:** 10.3389/fimmu.2025.1627514

**Published:** 2025-10-27

**Authors:** Daxina Bhatt, Yousef Alebrahim, Abdullah Shahzad, Lamiya Mohiyiddeen, Elizabeth Mann

**Affiliations:** ^1^ Faculty of Biology, Medicine & Health, University of Manchester, Manchester, United Kingdom; ^2^ Department of Reproductive Medicine, Saint Mary’s Hospital, Manchester, United Kingdom

**Keywords:** recurrent implantation failure, immune, immunology, fertility, cytokine, mediators

## Abstract

**Introduction:**

Embryo implantation is a complex process requiring a tightly regulated immunological dialogue at the maternal-embryonic interface. Disruptions in this dialogue, including alterations in immune cell function and cytokine production, have been implicated in implantation failure. This systematic review and meta-analysis aimed to quantitatively compare immune-related soluble mediators in the peripheral blood of women with unexplained recurrent implantation failure (RIF) and fertile controls.

**Methods:**

This systematic review was conducted according to PRISMA principles. A comprehensive search was conducted across Embase, MEDLINE, and the Cochrane Central Register of Controlled Trials. The primary outcome measure was the differential concentration of immune analytes in blood and tissue samples between women with recurrent implantation failure and fertile controls. Meta-analysis was performed for five peripheral blood cytokines (IFN-γ, IL-4, TNF-α, IL-2, IL-6).

**Results:**

Some 12 studies reporting on 1483 patients met the final inclusion criteria for the review. The meta-analysis revealed a statistically significant difference only for Interleukin-4 (IL-4), which was lower in women with RIF compared to controls (MD -0.0298, 95% CI: -0.0436 to -0.0159, p < 0.0001). No significant differences were found for IFN-γ, TNF-α, IL-2, or IL-6. Individual studies reported varied associations for other analytes, including lower levels of Angiopoietin-2, MMP-7, VEGF, FGF1, Glycodelin A, and MUC1, and higher levels of PDGF, TGF-β isoforms and CCL2, IL-2 in RIF cohorts. The overall certainty of the evidence was rated as low, due to concerns about study quality and heterogeneity in RIF definitions, control group selection, and laboratory methodologies.

**Conclusion:**

The review highlights that immune dysregulation is associated with RIF. In particular, IL-4 may play an important role although the clinical relevance of the small, measured difference is unclear. There is a need for international consensus on RIF definition, standardised methodological protocols, and large-scale prospective studies to validate potential immune biomarkers. Currently, there is insufficient evidence to support the routine use of peripheral blood cytokine levels as diagnostic markers for RIF or to guide immunomodulatory treatment.

**Systematic Review Registration:**

https://www.crd.york.ac.uk/prospero/, identifier PROSPERO 42024577277.

## Introduction

Embryo implantation is a key limiting factor in assisted reproductive technologies (ART). Recurrent implantation failure (RIF) is defined as the inability to achieve clinical pregnancy following transfer of multiple high-quality embryos over successive cycles (1), affecting a significant patient population. While standard investigations address uterine, endocrine, genetic, thrombophilia, paternal, or embryological factors, many RIF cases remain idiopathic.

Successful implantation requires complex immunological adaptations at both the maternal-fetal interface and systemically for fetal tolerance alongside pathogen defence (2). Key mechanisms include immune cell modulation, controlled inflammation, angiogenesis regulation, and tissue remodelling. Immune dysregulation is hypothesised as a major contributor to unexplained RIF (2). Successful implantation requires a delicate balance of both pro-inflammatory and anti-inflammatory responses at the maternal-fetal interface (3). A controlled, inflammatory response is vital. This response, driven by pro-inflammatory cytokines, initiates endometrial decidualisation, regulates extravillous trophoblast invasion, and promotes the angiogenesis needed to remodel uterine arteries (4). Simultaneously, immunoregulatory mechanisms must establish maternal tolerance to the semi-foreign conceptus, preventing its rejection (4).

This immune balance is mainly controlled by T-helper (Th) cell subsets and macrophage polarisation. A tightly regulated type 1 pro-inflammatory response involving T-helper type 1 (Th1) cells, classically activated (M1) macrophages, and cytokines including interferon-gamma (IFN-γ), tumour necrosis factor-alpha (TNF-α), granulocyte-macrophage colony-stimulating factor (GM-CSF), and interleukins IL-1, IL-2, and IL-12 is essential for tissue remodelling during implantation and placentation. This is balanced by a type 2 anti-inflammatory response mediated by T-helper type 2 (Th2) cells, regulatory T-cells (Tregs), and alternatively activated (M2) macrophages, which secrete cytokines such as interleukins IL-4, IL-6, IL-10, IL-13, and transforming growth factor-beta (TGF-β). This anti-inflammatory response establishes maternal tolerance, aids angiogenesis, and maintains tissue homeostasis. A precise equilibrium between these pathways is critical; excessive inflammation risks conceptus rejection, while too much tolerance can impair placental development (5, 6).

The maternal immune system undergoes a programmed temporal transition in its Th1/Th2 polarity throughout gestation. The peri-implantation period requires a Th1-dominant environment. This subsequently transitions to a predominantly Th2-skewed anti-inflammatory state during the second trimester, which is critical for supporting fetal growth and maintaining tolerance (7). The onset of labour is marked by a terminal pro-inflammatory shift back to Th1 dominance, facilitating the uterine contractions necessary for delivery (7). Consequently, the precise spatiotemporal regulation of this Th1/Th2 cytokine axis is a critical determinant of pregnancy outcome. Pathological dysregulation of this equilibrium is a primary etiological basis for a spectrum of reproductive and obstetric morbidities, including RIF, recurrent miscarriage, pre-eclampsia, and fetal growth restriction (4).

The immune involvement hypothesis has led to clinical use of immunological tests in RIF (8), including natural killer (NK) cells quantification, killer-cell immunoglobulin-like receptor (KIR)/human leukocyte antigen (HLA) genotyping, regulatory T cell (Treg) assessment, T-helper cell 1 (Th1)/Th2 cytokine ratios, and cytokine profiling. However, clinical utility of these assessments remains contentious due to insufficient validation, lack of standardised assay protocols and diagnostic thresholds, and inconsistent findings across studies without robust prospective data linking them to pregnancy outcomes. Consequently, major professional bodies and health authorities, including the Human Fertilisation and Embryology Authority (HFEA) in the UK and the European Society of Human Reproduction and Embryology (ESHRE) (9), currently advise against routine immune testing for RIF and categorise most immunological interventions as experimental.

Despite diagnostic uncertainty, some immunomodulatory therapies are often empirically used in RIF (10), based on presumed immune aetiology. While some trials have shown this approach may improve implantation rates, potentially by augmenting the local endometrial immune environment via cytokines such as TNF-α, IL-1ß and IFN-γ, these interventions are still considered experimental (10–12). ESHRE emphasises that such add-ons should not be offered routinely due to the lack of conclusive evidence of efficacy and safety, highlighting the need for a more complete understanding of the complex immunopathology of RIF and the risks associated with immune manipulation during early pregnancy (9).

Progress in understanding RIF immunopathology is hindered by heterogeneous and conflicting research findings. Although numerous studies report immune alterations, consistency is lacking. Crucially, a systematic synthesis of quantitative data for soluble mediators (e.g. cytokines, chemokines, growth factors, matrix metalloproteinases) involved in immune regulation, angiogenesis, and tissue remodelling is absent. Such analysis is needed for objective comparison between RIF patients and fertile controls, biomarker identification and resolving discrepancies.

Therefore, the primary objective of this study was to perform a systematic review and, where data permitted, meta-analysis to evaluate quantitative evidence comparing levels of immune-related soluble mediators in peripheral blood and uterine samples (tissue/fluid) between women with unexplained RIF (uRIF) and fertile controls. This quantitative synthesis aimed to summarise the current evidence, identify consistent findings or discrepancies, and define knowledge gaps to guide future research towards improved RIF diagnosis and management. To our knowledge, this is the first systematic review and meta-analysis to synthesise quantitative data across this spectrum of circulating mediators in RIF.

## Materials and methods

This systematic review and meta-analysis were conducted and reported in accordance with the Preferred Reporting Items for Systematic Reviews and Meta-Analysis (PRISMA) guidelines (13) and was prospectively registered on PROSPERO (CRD 42024577277). The study protocol was developed prior to data extraction and remained unchanged throughout the study period.

### Literature search

A comprehensive electronic literature search was conducted across Embase, MEDLINE, and the Cochrane Central Register of Controlled Trials (CENTRAL). The search date was 09 August 2024. Database interrogation, utilising the PubMed and NICE Healthcare Databases Advanced Search (HDAS) interfaces, employed a systematic search strategy incorporating Medical Subject Headings (MeSH), pertinent keywords, and Boolean operators “AND” and “OR”. Key search terms included “recurrent implantation failure”, “repeated implantation failure”, “immune profiling”, “comparison”, “cytokine”, “immune cell”, “assay”, “analyte”, and “biomarker”. Full search string combinations are detailed within the **Supplementary Material File**.

### Study selection and data extraction

Following the removal of duplicate records, two independent reviewers [DB and YA] conducted a two-stage screening process. Initially, titles and abstracts were assessed against pre-defined eligibility criteria. Subsequently, full-text articles meeting the initial criteria were reviewed. DB and YA independently extracted data into an electronic spreadsheet. These data related to study characteristics, patient demographics, and outcomes. Discrepancies arising during any stage were resolved through further review, discussion and, where necessary, consultation with a third reviewer [LM].

### Inclusion and exclusion criteria

Original studies were included if they met the following criteria: (i) reported comparative outcomes between patients with uRIF and controls; (ii) reported quantitative levels of the following immune factors (where provided) in blood and tissue samples: cytokines and chemokines, growth factors, angiogenic markers, and coagulation factors, adhesion molecules, and matrix metalloproteinases (MMPs) and inhibitors; (iii) were published in the English language; and (iv) were published on or after 1 January 2000.

Studies were excluded if they met any of the following criteria: (i) reported on patients with RIF and a history of genital tract abnormality, infectious or chronic autoimmune disease, or other conditions affecting systemic inflammation; (ii) did not provide a direct comparison between two or more groups; (iii) did not utilise controls with a history of pregnancy; (iv) utilised controls with a history of recurrent pregnancy loss (v) were not published as full-text manuscripts (encompassing abstracts, conference proceedings, and investigations with incomplete datasets); (vi) were not primary research articles (therefore excluding review articles, meta-analyses, case reports, and letters to the editor) or (vii) animal studies.

### Statistical analysis

Outcome measures were pre-specified *a priori*. The primary outcome measure was defined as the differential concentration of immune analytes between individuals with uRIF and control participants. When extracting data, units were recorded as pg/mL (which is equivalent to pg/mL). Meta-analysis was performed if at least 3 studies provided comparative results for a specific immune analyte. Meta-analysis was performed after conversion of summary statistics to mean and standard deviation, if median and range/inter-quartile range (IQR) were provided instead. As study populations exceeded n=25 in each case, mean and median were considered interchangeable (14). Standard deviation was calculated by either range*0.25 (14) or IQR/1.35 (15).

Meta-analyses were performed using the “meta” package in RStudio (Version 2024.12.1 + 563). For each analyte, a random-effects model (DerSimonian-Laird method) was used to pool the mean differences between RIF and control groups. Heterogeneity between studies was assessed using the I^2^ statistic and Cochran’s Q test. A p-value <0.05 was considered statistically significant.

### Assessment of risk of bias

To appraise the quality of the findings from the meta-analysis, the certainty of evidence was evaluated using principles described in the GRADE framework (16). This entailed an assessment of study quality using the AXIS appraisal system (17) with scores of 18 or higher suggesting good quality, 15–17 moderate quality, and 14 or less suggesting low quality. Heterogeneity was assessed using the I^2^ statistic and Cochran’s Q test, and publication bias through the generation and interpretation of Funnel Plots.

## Results

**Figure 1:**
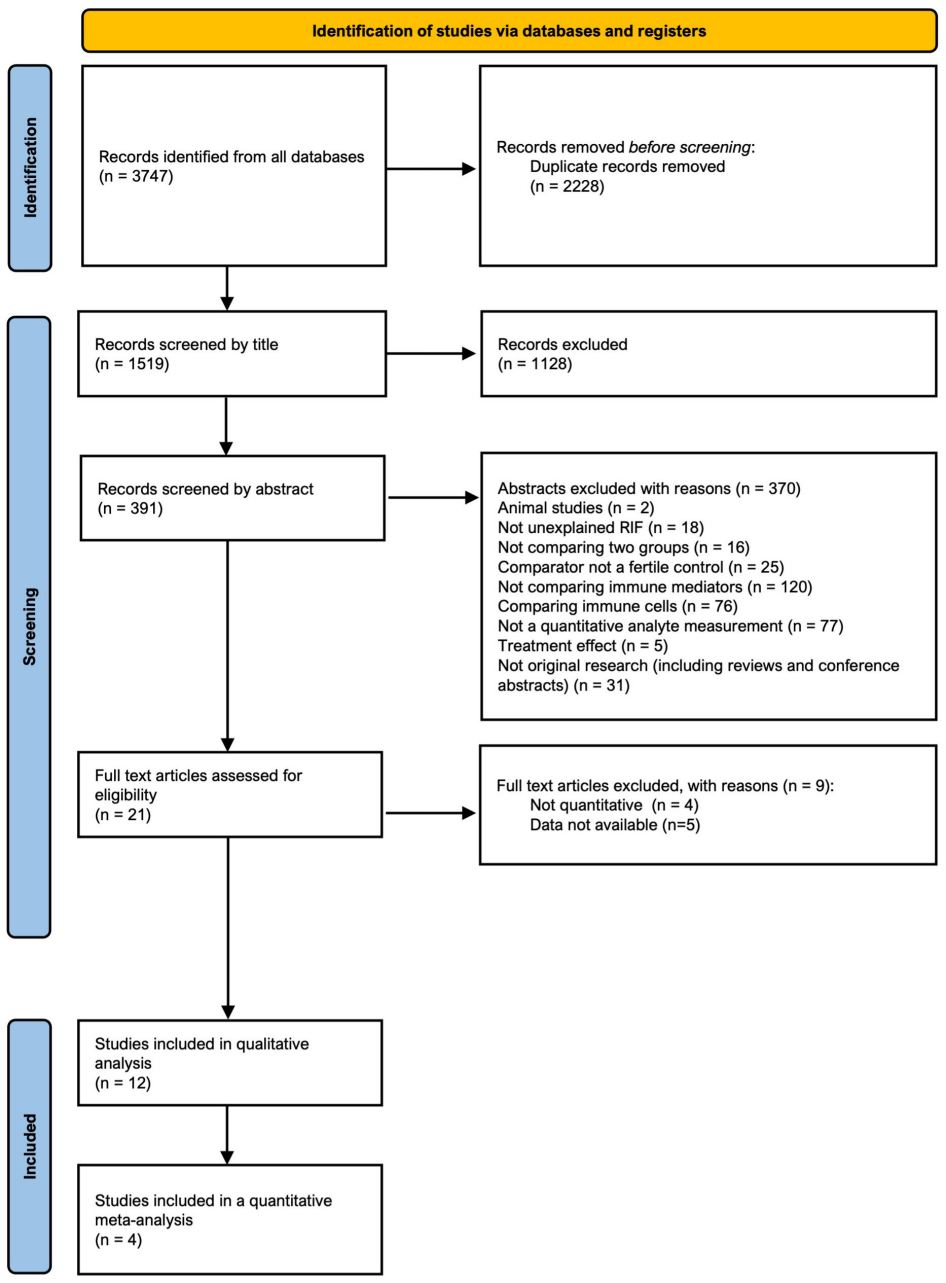
Preferred Reporting Items for Systematic Reviews and Meta-Analysis (PRISMA) flow diagram of identification, screening, eligibility, and inclusion phases of the systematic search for studies.

The search identified 1519 abstracts after de-duplication. 12 studies, reporting on 1483 patients met the final inclusion criteria for the review. All steps of the PRISMA search process with reasons for exclusion are provided in **Figure 1**.

### Study characteristics and design considerations

The 12 included studies were published between 2003 and 2024. All studies were observational in design and cross-sectional from the description of their methods. Study characteristics are presented in **Table 1**. Some 8 studies investigated the immune profile in blood samples alone, 3 in both blood and uterine tissue samples (including tissue and fluid aspirates) and 1 in uterine irrigation fluid alone. All studies compared a population of women with RIF to fertile controls.

**Table 1:** Study characteristics of included studies.

Authors	Publication year	Country	Recruitment year(s)	Study design	n total	n RIF	n Control	Control group
Abdulhaleem et al. (18)	2021	Iraq	2019	Case-Control	154	80	74	1) Healthy females, matched for age and BMI, and 2) females with IVF success
Benkhalifa et al. (19)	2021	Tunisia	2016-2017	Observational – subtype not specified	44	22	22	Successful embryo implantation in the 1^st^ cycle
Comins-Boo et al. (20)	2022	Spain	2017-2018	Observational – subtype not specified	55	24	31	Healthy women aged 20–45 who had at least 2 children and no history of miscarriage
Guo et al. (21)	2022	China	2020	Prospective Observational	70	41	29	Women achieving successful clinical pregnancy after the 1^st^ IVF/ICSI-ET cycle
Liang et al. (22)	2015	China	2013	Prospective Observational	59	34	25	Women <39 years old, basal FSH <10mIU/mL, antral follicle counts in both ovaries >7 and achieving successful clinical pregnancy after the 1^st^ IVF/ICSI-ET cycle
Nenonen et al. (23)	2024	Sweden	2007-2016	Retrospective Observational	55	29	26	Women with live birth after 1^st^ IVF cycle, combined with male factor infertility indication for IVF.
Kharamani et al. (24)	2024	Iran	–	Cross Sectional	800	400	400	Women who achieve pregnancy after 1^st^ embryo transfer.
Kalem et al. (25)	2017	Turkey	2014-2016	Cross Sectional	60	30	30	Multiparous women who have had 2 live births at term, and no history of miscarriage.
Gong et al. (26)	2017	China	2013-2015	Observational – subtype not specified	60	30	30	Women with live birth after 1^st^ IVF-ET cycle, combined with male factor infertility indication for IVF, and age <44 years old.
Bastu et al. (27)	2015	Turkey	–	Observational – subtype not specified	49	26	23	Women with 1 previous live birth, with no history of infertility or abortion.
Taheripanah et al. (28)	2017	Iran	2012	Prospective Observational	39	20	19	Fertile women of the same age range.
Inagaki et al. (29)	2003	Australia	–	Observational – subtype not specified	38	22	16	Multiparous women with a history of tubal sterilisation who were undergoing tubal anastamosis

RIF, recurrent implantation failure.

The definition of RIF varied across studies; RIF was defined by the number of transfers in some studies (≥3) and the number of cycles in others (≥2 or ≥3). The number of embryos transferred in each cycle as part of these definitions also varied across studies from ≥2 to ≥10. The cycle stage during which samples were collected was specified as mid-luteal in most studies (8/12) and not defined in the remainder. Control groups varied greatly in detail across the studies, but they all generally included women with successful pregnancies either with or without the use of assisted reproductive technology (**Table 1**).

There were a variety of laboratory-based methods employed to analyse samples across the 12 studies. Enzyme-linked immunosorbent assays (ELISA), and variations such as Sandwich Immunoassays were utilised for single analyte quantification, while multiplex assays, including Luminex and AimPlex, facilitated the simultaneous measurement of multiple analytes through bead-based flow cytometry. Cytometric bead arrays (CBA) offered another flow cytometry-based approach for multiplex analysis. Additionally, Western blotting and Immunohistochemistry (IHC) were utilised in those studies analysing tissue samples. Study design considerations are presented in **Table 2**.

**Table 2:** Definition, immune profiling methods and analytes in included studies.

Author	Definition of RIF: by number of embryos	Definition of RIF: by number of transfers	Definition of RIF: by number of cycles	Cycle stage	Sample type	Profiling method	Analytes reported - blood	Analytes reported - tissue/other fluid
Abdulhaleem et al. (18)	≥2	–	–	Not defined	Blood	ELISA	Angiopoietin 2	–
Benkhalifa et al. (19)	≥4	–	≥3	Mid-Luteal	Blood	ELISA	MMP1, MMP2, MMP3, MMP7, MMP9, TIMP1, TIMP2, LIF, VEGF, ICAM1, VCAM1	–
Comins-Boo et al. (20)	≥4	≥3	≥3	Not defined	Blood	Multiplex assay (Luminex)	Eotaxin, FGF2, IFNa, IFNg, IL1b, IL1ra, IL4, IL7, IL8, IL9, IL13, IL17a, IL18, CXCL10, CCL2, CCL4, PDGF, CCL5, TNFa, TRAIL, TGFb, TGFb2, TGFb3	–
Guo et al. (21)	≥4	–	≥3	Mid-Luteal	Blood	Multiplex assay (AimPlex)	IFNg, IL4, IL17a, TNFa, IL2, IL6, IL10, TNFb, GCSF, GMCSF	–
Liang et al. (22)	≥10	–	≥2	Mid-Luteal	Blood	Cytometric Bead Array	IFNg, IL1b, IL4, TNFa, TGFb1, IL6, IL10	–
Nenonen et al. (23)	–	≥3		Not defined	Blood	Sandwich Immunoassay	IFNg, IL1b, IL4, TNFa, IL2, IL6, Il10, IL12, IL13, IL18	–
Kharamani et al. (24)	≥4	–	≥3	Not defined	Blood	ELISA	FGF1	–
Kalem et al. (25)	≥4	–	≥3	Mid-Luteal	Blood	ELISA	CCL2	–
Gong et al. (26)	–	–	≥3	Mid-Luteal	Blood and Uterine Tissue	ELISA, Cytometric Bead Array and Immunohistochemistry	IL4, IL2, IL6, IL21	CXCR5, IL21
Bastu et al. (27)	≥4	–	≥3	Mid-Luteal	Blood and Uterine Tissue	ELISA and Western Blot	MUC1, GdA	GdA
Taheripanah et al. (28)	–	–	≥2	Mid-Luteal	Blood and Uterine cavity irrigation fluid	ELISA	GdA	GdA
Inagaki et al. (29)	≥10	–	–	Mid-Luteal	Uterine cavity irrigation fluid	ELISA	–	LIF, IFNg, IL1b, TNFa, IL10, MMP2, MMP9

CCL2, C-C Motif Chemokine Ligand 2; CXCL10, C-X-C Motif Chemokine Ligand 10, CXCR5, C-X-C Motif Chemokine Receptor 5; Eotaxin , Eosinophil Chemotaxis; FGF1, Fibroblast Growth Factor 1; FGF2, Fibroblast Growth Factor 2; GdA, Glycodelin A; GCSF, Granulocyte Colony-Stimulating Factor; IFN, Interferon Alpha; IFNg, Interferon Gamma, IL10, Interleukin 10; IL12, Interleukin 12; IL13, Interleukin 13; IL17a, Interleukin 17a, IL18 , Interleukin 18; IL1b, Interleukin 1 Beta; IL1ra, Interleukin 1 Receptor Antagonist; IL2 , Interleukin 2; IL21, Interleukin 21; IL4, Interleukin 4; IL6, Interleukin 6; IL7, Interleukin 7; IL8 , Interleukin 8; IL9, Interleukin 9;LIF , Leukaemia Inhibitory Factor; MMP1, Matrix Metalloproteinase 1; MMP2, Matrix Metalloproteinase 2; MMP3 , Matrix Metalloproteinase 3; MMP7, Matrix Metalloproteinase 7; MMP9, Matrix Metalloproteinase 9; MUC1, Mucin 1; TGFb1, Transforming Growth Factor Beta 1; TGFb3 , Transforming Growth Factor Beta 3; TIMP1, TIMP Metallopeptidase Inhibitor 1; TIMP2 ,TIMP Metallopeptidase Inhibitor 2; TNFa , Tumour Necrosis Factor Alpha; TNFb, Tumour Necrosis Factor Beta; VEGF, Vascular Endothelial Growth Factor.

Owing to a paucity of studies reporting on immune mediators in the local uterine compartment, the analysis of this review is centred on blood analytes. A total of four studies investigated the uterus, with two examining endometrial biopsies and the other two analysing mediator concentrations in uterine cavity irrigation fluid.

### Differential blood cytokine levels between RIF and controls

Some 8 studies reported differential blood concentrations between RIF patients and controls for a variety of immune markers. The immune markers analysed in each study are summarised in **Table 2**. Abdulhaleem et al. (18), in a case control study of 154 women demonstrated a significantly lower level of Angiopoietin-2 in patients with RIF compared to controls (2915pg/ml vs 3236pg/ml, p=0.007) and those with IVF success (2915pg/ml vs 3166pg/ml, p=0.009). Benkhalifa et al. (19), in a cohort study of 44 women, demonstrated lower levels of MMP-7 (119.97pg/ml vs 281.11pg/ml, p=0.03) and VEGF (30.93pg/ml vs 82.54pg/ml, p=0.022) in women with RIF as compared to controls. Comins-Boo et al. (20) studied the widest panel of immune analytes of all the studies, a total of 23 in an observational study of 55 patients; positive findings were higher levels of PDGF-BB (3498pg/mL vs 1659pg/mL, p<0.05), TGF-beta1 (25817pg/mL vs 15567pg/mL, p<0.05) and TGF-beta3 (249pg/mL vs 140pg/mL, p<0.01) in patients with RIF as compared to healthy controls. Guo et al. (21) demonstrated lower levels of IL-10 (2.18pg/mL vs 3.37 pg/mL, p=0.034), lower levels of G-CSF (5.36pg/mL vs 7.83pg/mL, p=0.033) and higher levels of IL-6 (3.61pg/mL vs 2.45pg/mL, p=0.042) in patients with RIF as compared to controls in a study of 70 women from China. They also investigated cytokine ratios, demonstrating higher ratios of IL-2/IL-10 as well as of IFN-γ/IL-10 in patients with RIF as compared to controls. Liang et al. (22) demonstrated significantly higher levels of IFN-γ, IL-6, IL-1b, IL-4, and TGF-b1 in 34 women with RIF as compared to 25 healthy controls. Furthermore, higher ratios of IFN-γ/IL-4, IFN-γ/IL-10, IFN-γ/TGF-b1, IL-6/IL-10, IL-6/TGF-b1, and IL-1b/TGF-b1 were seen in the RIF cohort. Nenonen et al. (23) demonstrated lower levels of IFN-γ (5.45pg/mL vs 5.78pg/mL, p=0.001) and IL-2 (0.26pg/mL vs 0.45 pg/mL) in RIF patients compared to controls. Kharamani et al. (24) performed the largest study included in this review: a comparative analysis of 400 RIF patients and 400 controls. They focussed on Fibroblast Growth Factors alone and demonstrated significantly lower levels in the blood of women with RIF (17pg/mL vs 23pg/mL, p=0.008). Kalem et al. (25) also focussed on a single analyte, CCL2, key for monocyte recruit to tissue, and demonstrated higher levels in RIF patients (and those with recurrent miscarriage) than controls (29.8pg/mL vs 22.7pg/mL, p<0.001). Gong et al. (26) demonstrated increased levels of IL-21 (21.8pg/mL vs 14.01pg/mL, p<0.05) and IL-6 (14.45pg/mL vs 9.87pg/mL, p<0.05) as compared to controls. Bastu et al. (27) studied the role of two glycoproteins: Mucin 1 and Glycodelin A. Both were found to be significantly lower in patients with RIF as compared to controls. Finally, Taheripanah et al. (28) demonstrated lower glycodelin A concentrations in the blood of RIF patients than controls (30.1 ng/mL vs 44.5 ng/mL, p<0.001).

### Quantitative pooled analysis

Five blood analytes were reported in at least 3 studies and so enabled quantitative review with meta-analysis; they were IFN-γ, IL-4, TNF-alpha, IL-2 and IL-6. Studies included in the meta-analysis were Comins Boo et al. (20), Guo et al. (21), Nenonen et al. (23) and Gong et al. (26). One additional study (Liang et al. (22)) was identified as having cytokine concentration levels many orders of magnitude higher than those reported in other studies. The inclusion of this study would have resulted in a failure of the meta-analysis model to converge, indicating an undue influence and violation of assumptions of the meta-analysis. Consequently, this study was excluded from all meta-analyses to ensure the validity and stability of the results.

#### Meta-analysis of interferon-gamma levels

**Figure 2:**
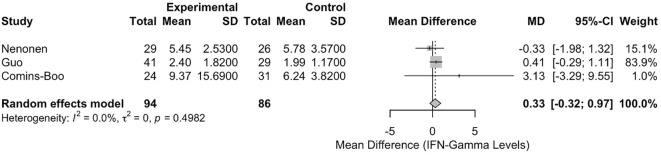
Forest plot of studies reporting on IFN-gamma levels in RIF versus controls.

The meta-analysis of IFN-γ levels included three studies [Comins-Boo (20), Guo (21), and Nenonen (23)], encompassing a total of 180 participants (94 in the RIF group and 86 in the control group). The pooled mean difference (MD) between the RIF and control groups was 0.3255 (95% CI: -0.3168, 0.9677). This result was not statistically significant, p = 0.3206 (**Figure 2**). Heterogeneity among the studies was low, with an I² statistic of 0.0% (95% CI: 0.0%, 89.6%) and a tau² of 0. The test for heterogeneity was not significant (Q = 1.39, df = 2, p = 0.4982).

#### Meta-analysis of interleukin-4 levels

**Figure 3:**
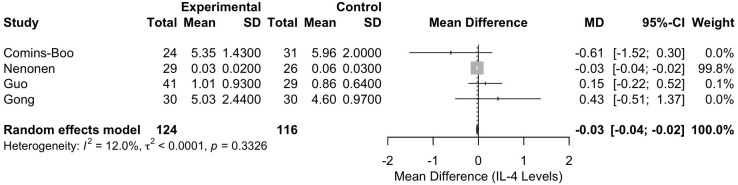
Forest plot of studies reporting on IL-4 levels in RIF versus controls.

The meta-analysis of IL-4 levels included four studies [Comins-Boo (20), Guo (21), Nenonen (23), and Gong (26)], with a total of 240 participants (124 in the RIF group and 116 in the control group). The pooled MD between the RIF and control groups was -0.0298 (95% CI: -0.0436, -0.0159). This result was statistically significant, p < 0.0001, indicating a lower level of IL-4 in the RIF group (**Figure 3**). Heterogeneity among the studies was low, with an I² statistic of 12.0% (95% CI: 0.0%, 86.5%) and a tau² of less than 0.0001. The test for heterogeneity was not significant (Q = 3.41, df = 3, p = 0.3326).

#### Meta-analysis of tumour necrosis factor-alpha levels

**Figure 4:**
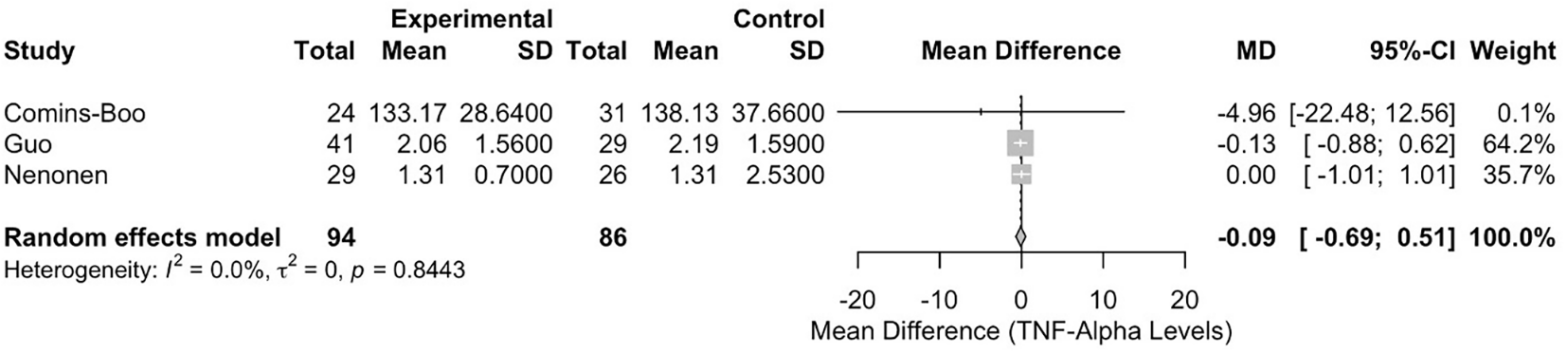
Forest plot of studies reporting on TNF-alpha levels in RIF versus controls.

The meta-analysis of TNF-α levels included three studies [Comins-Boo (20), Guo (21), and Nenonen (23)], with 180 participants (94 in the RIF group and 86 in the control group). The pooled MD between the RIF and control groups was -0.0892 (95% CI: -0.6902, 0.5117). This result was not statistically significant, p = 0.7710 (**Figure 4**). Heterogeneity among the studies was low, with an I² statistic of 0.0% (95% CI: 0.0%, 89.6%) and a tau² of 0. The test for heterogeneity was not significant (Q = 0.34, df = 2, p = 0.8443).

#### Meta-analysis of interleukin-2 levels

**Figure 5:**
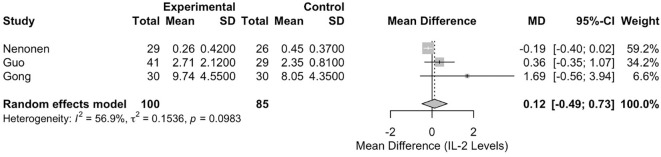
Forest plot of studies reporting on IL-2 levels in RIF versus controls.

The meta-analysis of IL-2 levels included three studies (Gong (26), Guo (21), and Nenonen (23)), with 185 participants (100 in the RIF group and 85 in the control group). The pooled MD between the RIF and control groups was 0.1224 (95% CI: -0.4901, 0.7349). This result was not statistically significant, p = 0.6952 (**Figure 5**). Heterogeneity among the studies was moderate, with an I² statistic of 56.9% (95% CI: 0.0%, 87.7%) and a tau² of 0.1536. The test for heterogeneity was not statistically significant (Q = 4.64, df = 2, p = 0.0983).

#### Meta-analysis of interleukin-6 levels

**Figure 6:**
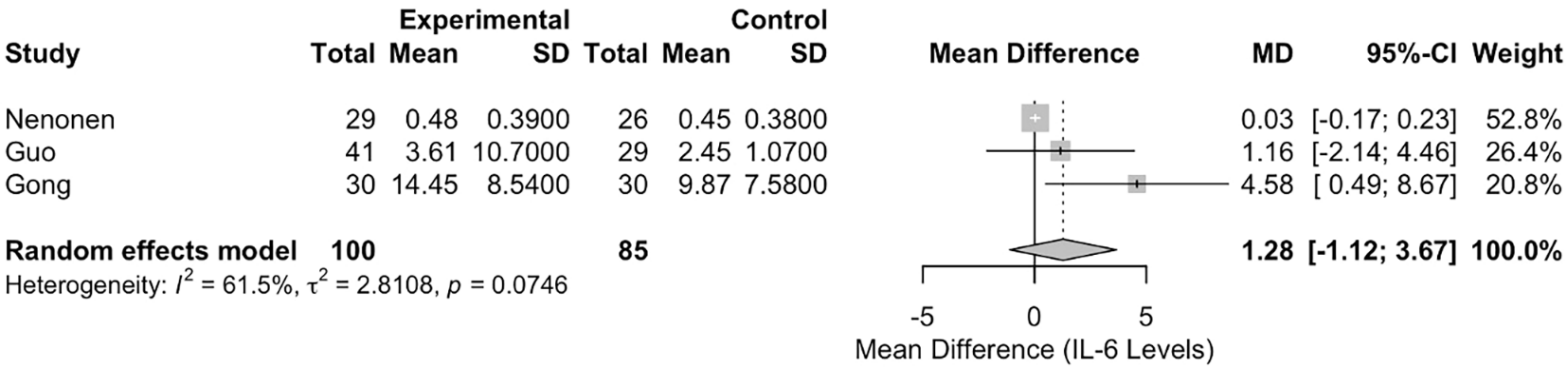
Forest plot of studies reporting on IL-6 levels in RIF versus controls.

The meta-analysis of IL-6 levels included three studies (Gong (26), Guo (21), and Neonen (23)), with 185 participants (100 in the RIF group and 85 in the control group). The pooled MD between the RIF and control groups was 1.2752 (95% CI: -1.1168, 3.6673). This result was not statistically significant, p = 0.2961 (**Figure 6**). Heterogeneity among the studies was moderate, with an I² statistic of 61.5% (95% CI: 0.0%, 89.0%) and a tau² of 2.8108. The test for heterogeneity was not statistically significant (Q = 5.19, df = 2, p = 0.0746).

### Assessment of certainty and quality of evidence from studies included in meta-analysis

Heterogeneity was not significant in any of the 5 meta-analyses performed, as determined by the I^2^ statistic and Cochrane Q test. Publication bias was not a concern given the appearances of Funnel Plots for the 5 meta-analyses performed (**Supplementary Material**). A statistical test for publication bias using Egger’s test was not possible given the low number of studies in each meta-analysis.

In terms of study quality, the 4 studies were assessed using the AXIS appraisal system, which was designed specifically for cross-sectional studies (17). There were concerns raised by the AXIS tool for study quality across the 4 studies. 3 studies [Nenonen (23), Guo (21) and Comins-Boo (20)] were graded as “moderate” quality (score range 15–17 inclusive) and 1 [Gong (26)] as “poor” quality (score 14 or less). The scoring sheet is provided in the **Supplementary Material**.

There was inconsistency between studies in terms of definitions for both RIF and the nature of the controls. Furthermore, as described earlier, 1 study [Liang et al. (22)] was also excluded from meta-analysis due to the concerns with unexplained magnitude of result compared to other studies, for absolute cytokine concentrations.

Overall, there would be low confidence in the findings from the meta-analysis when all the considerations above are considered within a GRADE framework.

## Discussion

### Principal findings

This systematic review and meta-analysis represent, to our knowledge, the first attempt to quantitatively synthesise the evidence comparing specific immune mediators (encompassing cytokines, angiogenic factors, and tissue remodelling factors) between women with unexplained recurrent implantation failure (uRIF) and fertile controls. Our analysis of twelve observational studies published since 2000 revealed significant heterogeneity in study design, RIF definitions, control group selection, and laboratory methodologies. Despite pooling data for five peripheral blood cytokines (IFN-γ, IL-4, TNF-α, IL-2, IL-6) reported in three or more studies, only Interleukin-4 (IL-4) demonstrated a statistically significant difference, being lower in women with RIF compared to controls (MD -0.0298, 95% CI: -0.0436 to -0.0159, p < 0.0001). Meta-analyses for peripheral IFN-γ, TNF-α, IL-2, and IL-6 levels did not reveal statistically significant differences between the groups. Individual studies reported varied associations for a wide array of other analytes in both blood and uterine samples (including tissue and fluid), such as lower levels of Angiopoietin-2, MMP-7, VEGF, FGF1, Glycodelin A, and MUC1, and higher levels of PDGF, TGF-β isoforms, CCL2 and IL-21 in RIF cohorts. However, due to the limited number of studies investigating each specific analyte and methodological inconsistencies, further quantitative pooling was largely prevented. The overall certainty of the evidence derived from the meta-analysed studies was assessed as low using the GRADE framework (16), primarily due to concerns regarding study quality and clinical heterogeneity.

### Synthesis of evidence

We observed significantly lower peripheral IL-4 concentrations in women with RIF. This finding is important, especially considering the low statistical heterogeneity in this specific meta-analysis (I²=12.0%). IL-4 is a key Th-2 cytokine, typically associated with promoting immune tolerance crucial for embryo implantation, in contrast to pro-inflammatory Th1 responses implicated in implantation failure (13). Reduced systemic IL-4 levels could therefore suggest a shift away from this required state of immune tolerance. However, this observation is not consistent across all individual studies (e.g., Liang et al. (22), excluded from meta-analysis due to data anomalies) and challenges the established Th1/Th2 model. The biological importance of this single systemic finding remains uncertain, particularly whether it accurately reflects the critical immune dialogue at the feto-maternal interface within the endometrium. The absolute serological level difference of IL-4 between RIF and control patients was small and therefore raises the question of whether there is any clinical significance despite there being statistical significance.

Conversely, the lack of significant pooled differences for peripheral IFN-γ, TNF-α, IL-2, and IL-6 is also informative. Although individual studies in our review and the wider literature report associations between these cytokines and RIF [e.g. Guo et al. (21), Liang et al. (22), Nenonen et al. (23)], our meta-analysis indicates no consistent, statistically robust difference in their systemic levels based on current quantitative evidence. This result might reflect several factors: (i) a true absence of a strong systemic association; (ii) inadequate statistical power due to the small number of studies; (iii) the masking effect of substantial clinical and methodological heterogeneity; or (iv) peripheral blood measurements may be poor indicators of the dynamic local immune environment within the endometrium during implantation. The moderate heterogeneity for IL-2 (I²=56.9%) and IL-6 (I²=61.5%), although not statistically significant (perhaps due to low study numbers), suggests underlying variability between studies that requires cautious interpretation.

The varied findings from individual studies across diverse analytes (e.g., VEGF, FGF, MMP-7, Glycodelin A (GdA), CCL2) highlight the complex nature of immune pathways involved in RIF. Reduced levels of factors involved in angiogenesis (e.g., Ang-2, VEGF) or endometrial receptivity (e.g., GdA, MUC1), along with changes in tissue remodelling enzymes (MMPs) and growth factors (e.g., PDGF, TGF-β), could reasonably contribute to implantation failure.

Due to the limited number of studies, our review was unable to provide a detailed interpretation of findings from analyses of endometrial tissue or uterine fluid. Only four studies investigated the uterine compartment: two examined endometrial tissue (26, 27), and two analysed uterine fluid (28, 29), with findings related to markers such as lower glycodelin-A (GdA), higher IL-21/CXCR5, and altered matrix metalloproteinases (MMPs) and cytokines. Although data on endometrial mediators in RIF are scarce, significant research has been conducted in other clinical contexts. Notably, a recent randomised controlled trial by Lédée et al. demonstrated improved live birth rates in patients undergoing their first embryo transfer following personalised immunomodulatory interventions (30). These interventions were guided by endometrial immune profiling, specifically assessing mediators related to natural killer (NK) cell function (e.g., IL-15 and IL-18). This approach highlights a promising area for future investigation in RIF patients.

The endometrial compartment offers the most direct insight into the maternal-fetal immunological interface. However, the practicality and feasibility of investigating this compartment, especially during the narrow window of implantation, remains a topic of debate. While systemic blood measurements may not perfectly mirror the immunological status of the uterine niche, they could provide valuable insights into a general inflammatory state that either contributes to or results from implantation failure, thereby perpetuating a non-receptive endometrial environment (31).

### Strengths and limitations

This systematic review followed a rigorous protocol, adhering to PRISMA guidelines (13) and prospective PROSPERO registration, enhancing transparency and minimising reporting bias. We used a comprehensive search strategy across multiple databases. The review focussed on studies reporting quantitative analyte levels, allowing objective comparison and meta-analysis where feasible. Inclusion and exclusion criteria were defined precisely to target unexplained RIF and appropriate fertile controls, aiming to isolate immunological factors. Data extraction and study selection were performed independently by two reviewers to reduce error. We formally assessed the risk of bias within studies using the AXIS tool (17) and evaluated the overall certainty of meta-analysis evidence using GRADE principles (16), providing a critical perspective on the findings’ reliability.

However, several important limitations affect the interpretation and generalisability of our findings. First, all included primary studies were observational, preventing the establishment of causality. Second, there was substantial heterogeneity in RIF definitions (varying numbers of failed cycles/embryos) and control group characteristics, introducing a risk of confounding. Historically, RIF definitions have varied greatly (e.g., ≥2 failed cycles or ≥3 failed cycles) (1), while more recent ESHRE guidelines propose an individualised approach based on cumulative predicted chance of implantation (9). The variability in RIF definitions across studies makes direct comparison and quantitative synthesis difficult, and we accept this as a limitation of meta-analysing such data.

Third, methodological variability was considerable, including different sample types (blood, tissue, fluid), diverse assays (ELISA, multiplex, IHC, Western Blot) with varying sensitivity and specificity, and inconsistent reporting of sample timing (though most targeted the mid-luteal phase).

Fourth, the small number of studies per meta-analysis (n=3 or 4) limited statistical power to detect subtle differences and prevented robust assessment of publication bias or meaningful subgroup analyses. Furthermore, this review was restricted to specific soluble mediators and did not capture the full complexity of immunological assessment, which often includes cellular components (e.g., NK cell counts/activity, Treg populations) or genetic factors (e.g., KIR-HLA interactions). Finally, the reliance on peripheral blood studies may not accurately reflect crucial local events within the endometrium. The few studies examining tissue or uterine fluid, although possibly more relevant, were limited in number and used varied techniques. The quality assessment revealed moderate-to-poor methodological rigour in many studies, further reducing confidence in the pooled estimates, as reflected by the overall ‘Low’ GRADE assessment.

#### Implications for clinical practice

Based on this quantitative synthesis, there is currently insufficient robust evidence to support the routine use of peripheral blood levels of IFN-γ, IL-4, TNF-α, IL-2, or IL-6 as diagnostic markers for RIF or to guide empirical immunomodulatory treatment. The finding of lower systemic IL-4 in RIF warrants further investigation but needs substantial independent validation in well-designed studies before any clinical application can be considered. The numerous conflicting or isolated findings for other analytes reinforce the conclusion that a reliable, validated immune biomarker panel for RIF is not yet available. Clinicians should continue to exercise caution, consistent with guidance from bodies such as ESHRE (9), regarding the empirical use of unvalidated immunological tests and immunotherapies with unproven efficacy. Decisions about immunological testing and treatment should ideally occur within a research context or after careful consideration of the limited evidence and associated harms.

#### Implications for future research

The limitations identified highlight directions for future research. There is an urgent need for international consensus on the definition of uRIF and standardised criteria for selecting control groups. Methodological standardisation is essential, covering protocols for sample collection (timing, procedures), processing, assay selection (addressing variability, especially in multiplex platforms), and reporting units. Future work should prioritise large-scale, prospective cohort studies with detailed clinical phenotyping and longitudinal sample collection, ideally tracking immune profiles across cycle phases or treatment.

Independent validation of candidate analytes from single studies (e.g., GdA, Ang-2, TGF-βs) in separate, well-characterised cohorts is essential. Finally, moving beyond correlational studies to research investigating the functional consequences of observed immune alterations is necessary to determine underlying mechanisms and identify valid therapeutic targets.

## Conclusion

This systematic review and meta-analysis have highlighted that immune dysregulation is associated with uRIF. Studies have demonstrated a range of both pro- and anti- inflammatory immune mediators that significantly vary in concentration between women with RIF and fertile controls. However, studies are currently small, largely cross-sectional in design and not of high quality. Upon meta-analysis of 4 studies that reported differential blood concentrations of IL-4, it was shown to be present in lower concentrations in women with RIF as compared to controls. Overall confidence in this finding is low and further work is required to investigate his finding further with longitudinal studies. No consistent systemic pattern involving IFN-γ, TNF-α, IL-2, or IL-6 emerged.

Interpretation of findings from this review are largely limited by varied definitions of RIF and selection of control populations. Standardised protocols and reporting will aid the pooling of future data and provide clinical relevance to the findings of these studies. This is required before any immune biomarkers implicated in RIF can be considered potential targets for translation into clinically useful diagnostic tools and targeted therapies.

## Data Availability

The raw data supporting the conclusions of this article will be made available by the authors, without undue reservation.
