# Supplementary material for: Comparing the circulating immune profile of women with and without recurrent implantation failure: a systematic review and meta-analysis
Source: Front Immunol. 2025 Oct 27;16:1627514. doi: 10.3389/fimmu.2025.1627514 (PMC12597810; doi:10.3389/fimmu.2025.1627514)

Supplementary Material

1. **Systematic review search string combinations and results, by database.**

**Pubmed Medline**

1. ("Recurrent implantation failure" OR "repeated implantation failure") AND (“immune” OR "immune profiling" OR "immune profile" OR “assay”) n=234
2. ("Recurrent implantation failure" OR "repeated implantation failure") AND (“biomarker” OR “immune cell”) n=51
3. ("Recurrent implantation failure" OR "repeated implantation failure") AND (“cytokine” OR “chemokine” OR “analyte”) n=55
4. ("Recurrent implantation failure" OR "repeated implantation failure") AND (“serum” OR “peripheral blood”) n = 134
5. ("Recurrent implantation failure" OR "repeated implantation failure") AND (“endometrial biopsy” OR “uterine biopsy” OR “uterine fluid”) n = 79
6. ("Recurrent implantation failure" OR "repeated implantation failure") AND (“T cell” OR “B cell” OR “macrophage” OR “natural killer cell” OR “monocyte” OR “dendritic cell” OR “neutrophil” OR “basophil” OR “eosinophil” OR “innate lymphoid cell”) n = 56
7. ("Recurrent implantation failure" OR "repeated implantation failure") AND (“comparison” OR “compared”) AND (“control” OR “fertile control”) n = 247

**Embase Ovid**

1. (("Recurrent implantation failure" or "repeated implantation failure") and ("immune" or "immune profiling" or "immune profile" or "assay")) n=561
2. (("Recurrent implantation failure" or "repeated implantation failure") and ("biomarker" or "immune cell")) n=92
3. (("Recurrent implantation failure" or "repeated implantation failure") and ("cytokine" or "chemokine" or "analyte")) n=260
4. (("Recurrent implantation failure" or "repeated implantation failure") and ("serum" or "peripheral blood")) n=357
5. (("Recurrent implantation failure" or "repeated implantation failure") and ("endometrial biopsy" or "uterine biopsy" or "uterine fluid")) n=218
6. (("Recurrent implantation failure" or "repeated implantation failure") and ("T cell" or "B cell" or "macrophage" or "natural killer cell" or "monocyte" or "dendritic cell" or "neutrophil" or "basophil" or "eosinophil" or "innate lymphoid cell")) n=390
7. (("Recurrent implantation failure" or "repeated implantation failure") and ("comparison" or "compared") and ("control" or "fertile control")) n=501

**CENTRAL**

1. ("Recurrent implantation failure" OR "repeated implantation failure") AND (“immune” OR "immune profiling" OR "immune profile" OR “assay”) n= 50
2. ("Recurrent implantation failure" OR "repeated implantation failure") AND (“biomarker” OR “immune cell”) n= 13
3. ("Recurrent implantation failure" OR "repeated implantation failure") AND (“cytokine” OR “chemokine” OR “analyte”) n= 54
4. ("Recurrent implantation failure" OR "repeated implantation failure") AND (“serum” OR “peripheral blood”) n = 96
5. ("Recurrent implantation failure" OR "repeated implantation failure") AND (“endometrial biopsy” OR “uterine biopsy” OR “uterine fluid”) n = 42
6. ("Recurrent implantation failure" OR "repeated implantation failure") AND (“T cell” OR “B cell” OR “macrophage” OR “natural killer cell” OR “monocyte” OR “dendritic cell” OR “neutrophil” OR “basophil” OR “eosinophil” OR “innate lymphoid cell”) n = 26
7. ("Recurrent implantation failure" OR "repeated implantation failure") AND (“comparison” OR “compared”) AND (“control” OR “fertile control”) n = 231
8. **Funnel Plots for assessment of Publication Bias**


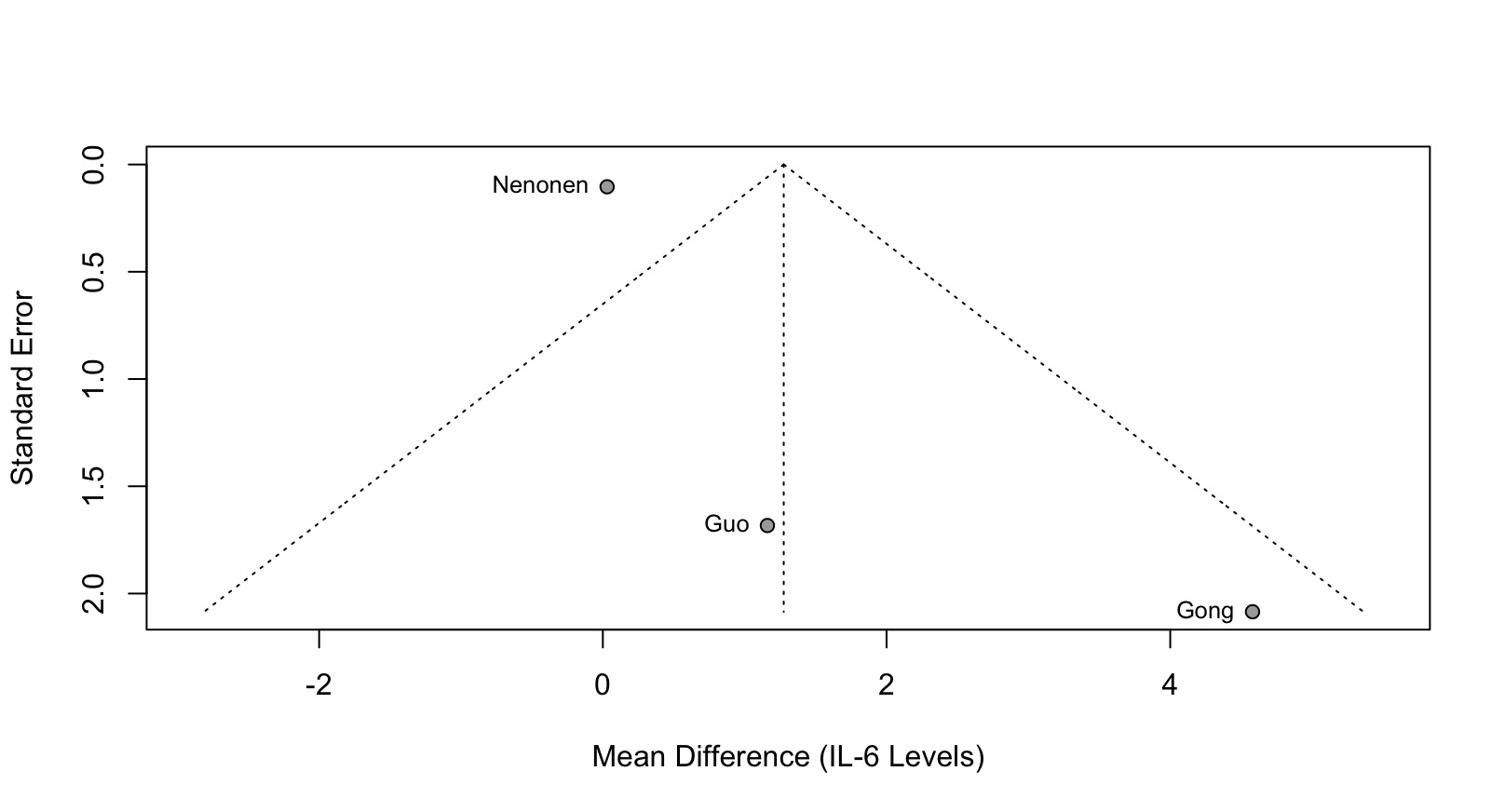


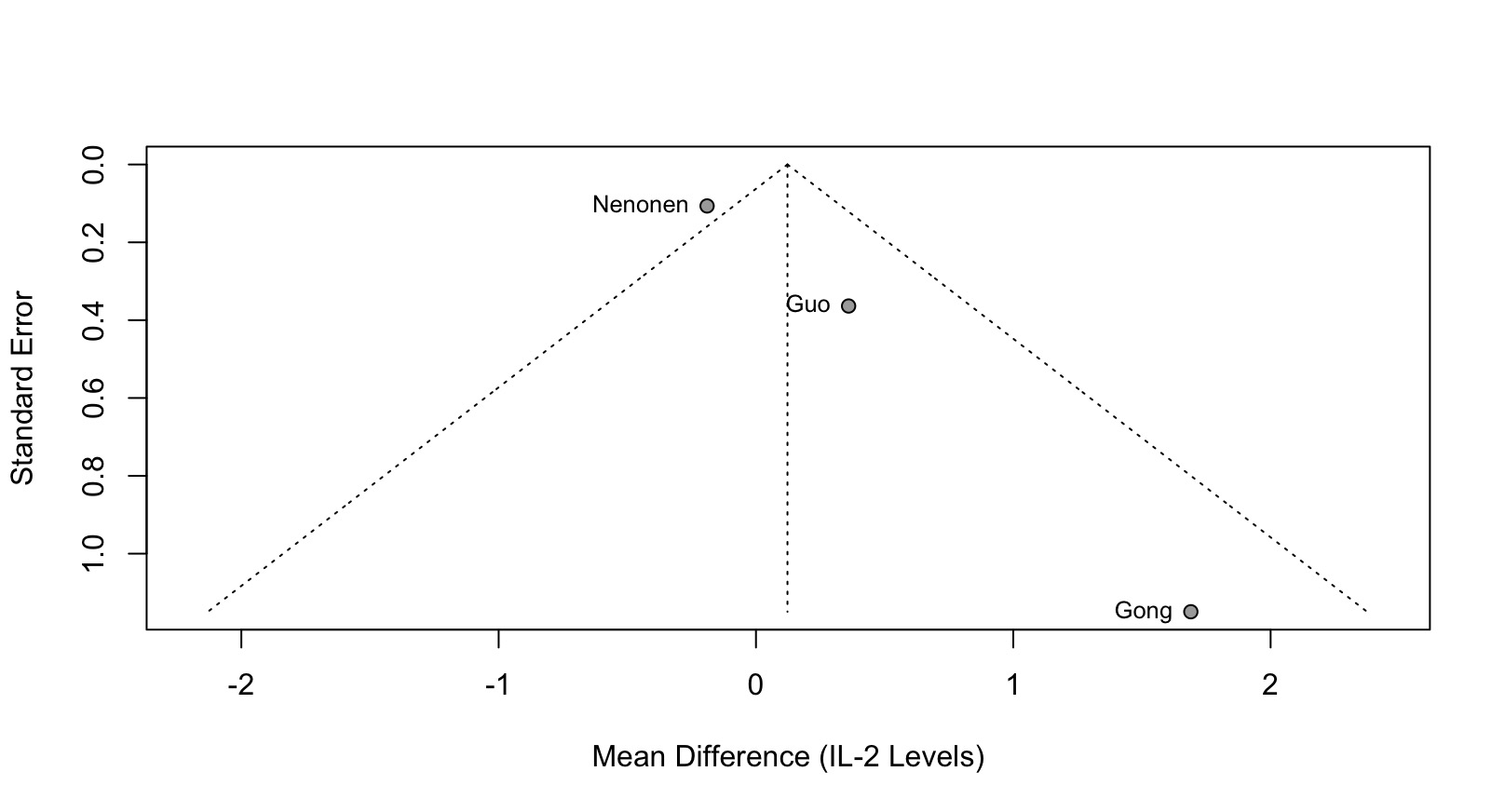


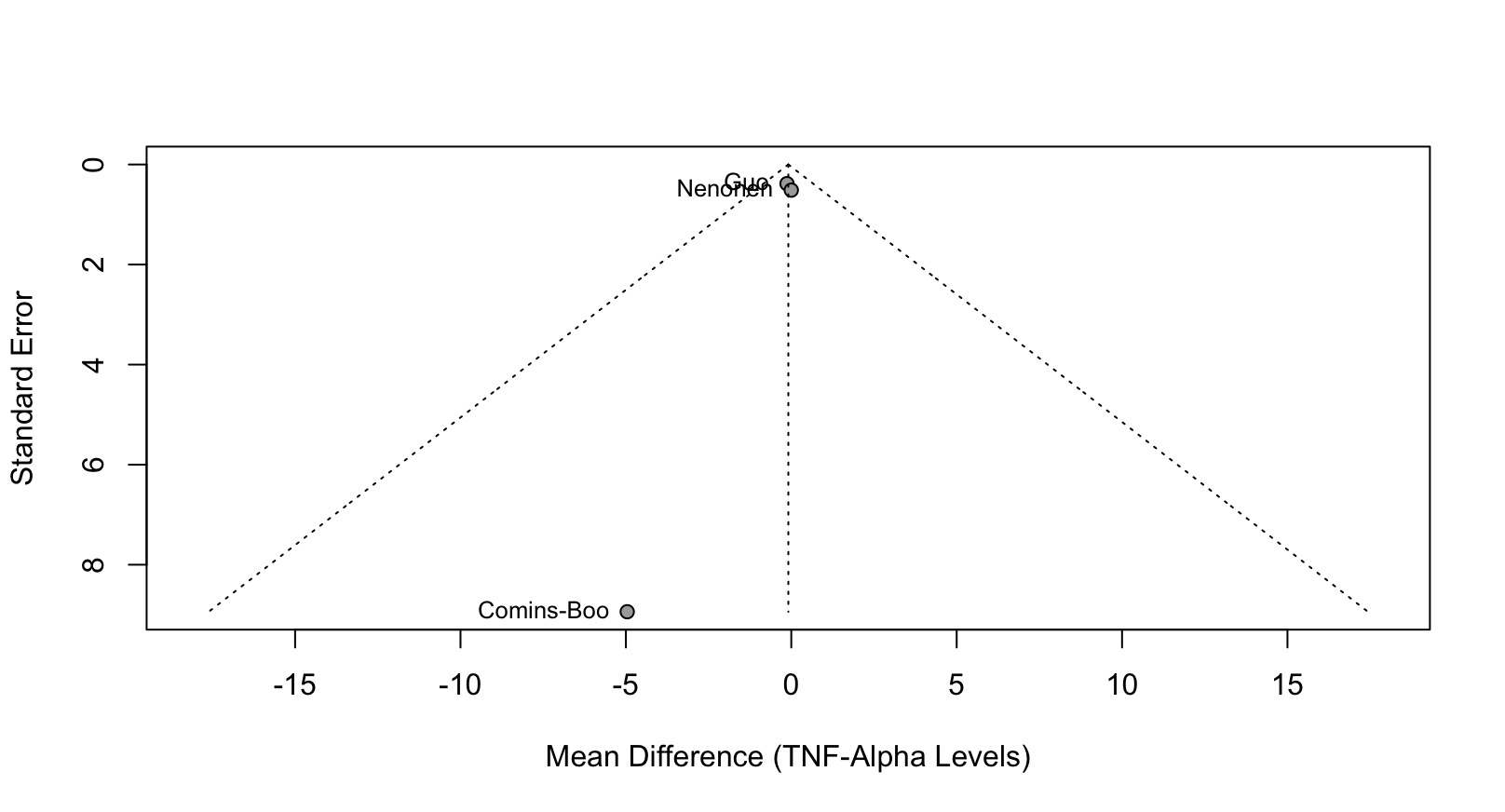


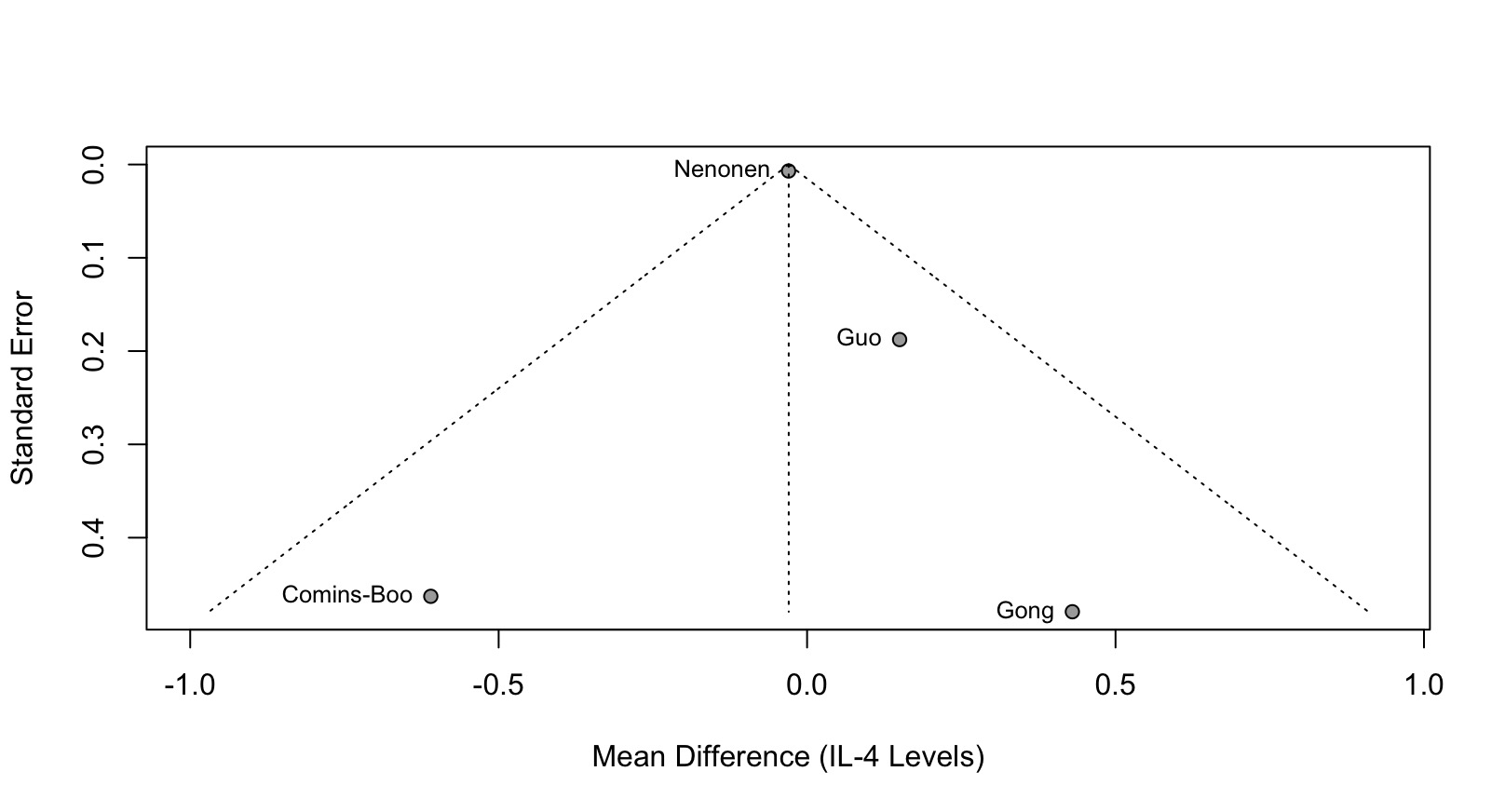


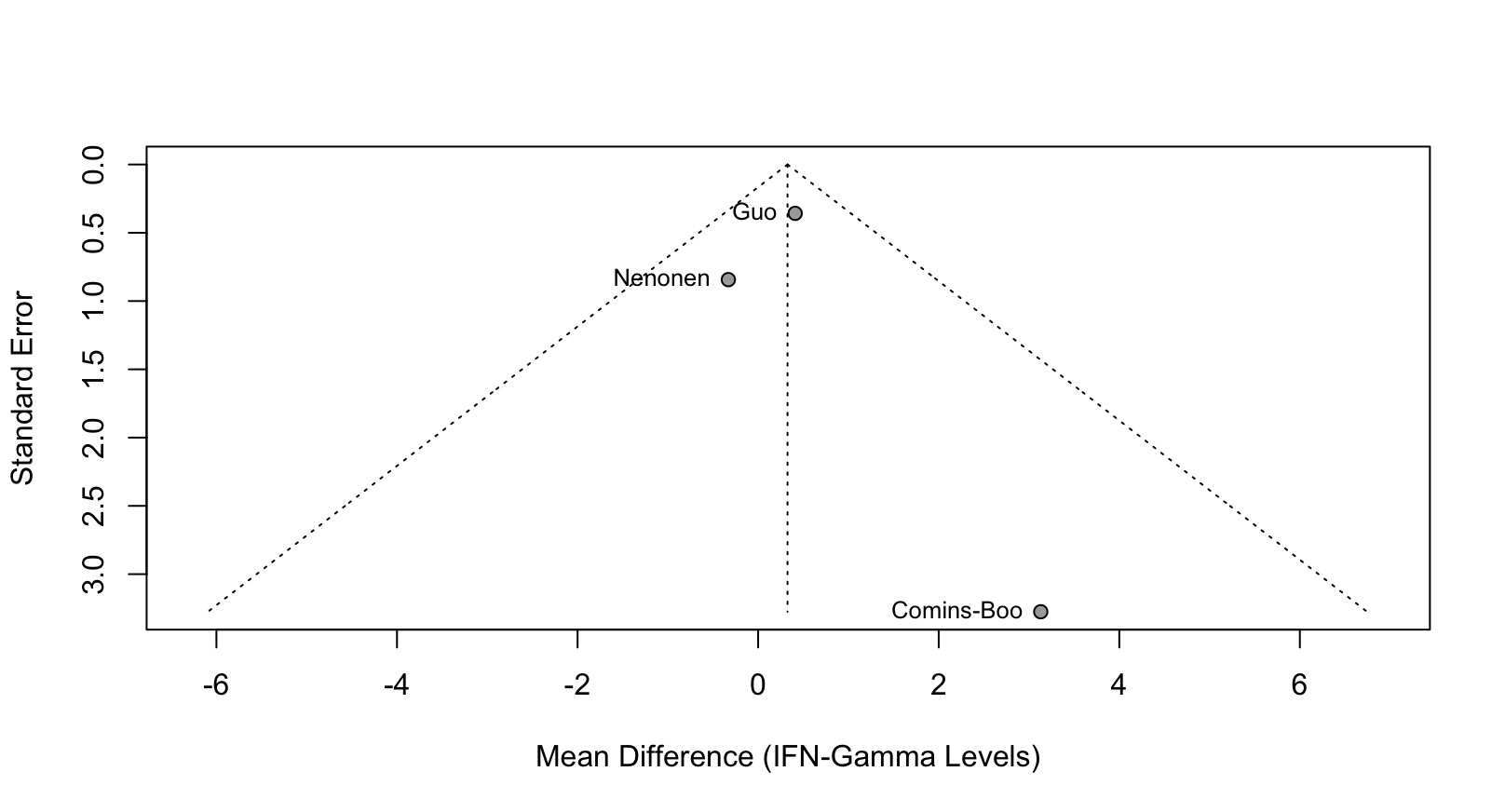


1. **AXIS tool for appraising the quality of the 4 studies included in meta-analysis**

*Score of 1 for “Yes/No concerns”*

*Score of 0 for “No/Unclear/Some concerns”*

|  | | **Comins-Boo et al** | **Gong et al** | **Guo et al** | **Neonen et al** |
| --- | --- | --- | --- | --- | --- |
| ***Introduction*** | |  |  |  |  |
| 1 | Were the aims/objectives of the study clear? | **1** | **1** | **1** | **1** |
| ***Methods*** | |  |  |  |  |
| 2 | Was the study design appropriate for the stated aim(s)? | **1** | **1** | **1** | **1** |
| 3 | Was the sample size justified? | **0** | **0** | **0** | **0** |
| 4 | Was the target/reference population clearly defined? (Is it clear who the research was about?) | **1** | **1** | **1** | **1** |
| 5 | Was the sample frame taken from an appropriate population base so that it closely represented the target/reference population under investigation? | **1** | **1** | **1** | **1** |
| 6 | Was the selection process likely to select subjects/participants that were representative of the target/reference population under investigation? | **0** | **0** | **0** | **1** |
| 7 | Were measures undertaken to address and categorise non-responders? | **0** | **0** | **0** | **0** |
| 8 | Were the risk factor and outcome variables measured appropriate to the aims of the study? | **1** | **1** | **1** | **1** |
| 9 | Were the risk factor and outcome variables measured correctly using instruments/measurements that had been trialled, piloted or published previously? | **1** | **1** | **1** | **1** |
| 10 | Is it clear what was used to determined statistical significance and/or precision estimates? (eg, p values, CIs) | **1** | **1** | **1** | **1** |
| 11 | Were the methods (including statistical methods) sufficiently described to enable them to be repeated? | **1** | **1** | **1** | **1** |
| ***Results*** | |  |  |  |  |
| 12 | Were the basic data adequately described? | **1** | **1** | **1** | **1** |
| 13 | Does the response rate raise concerns about non-response bias? | **1** | **1** | **1** | **1** |
| 14 | If appropriate, was information about non-responders described? | **0** | **0** | **0** | **0** |
| 15 | Were the results internally consistent? | **1** | **1** | **1** | **1** |
| 16 | Were the results for the analyses described in the methods, presented? | **1** | **1** | **1** | **1** |
| ***Discussion*** | |  |  |  |  |
| 17 | Were the authors’ discussions and conclusions justified by the results? | **1** | **1** | **1** | **1** |
| 18 | Were the limitations of the study discussed? | **1** | **0** | **0** | **1** |
| ***Other*** | |  |  |  |  |
| 19 | Were there any funding sources or conflicts of interest that may affect the authors’ interpretation of the results? | **0** | **0** | **0** | **0** |
| 20 | Was ethical approval or consent of participants attained? | **1** | **1** | **1** | **1** |
| TOTAL SCORE (/20) | | **15** | **14** | **15** | **16** |

1. **Sensitivity Analysis**

A score of less than 15 on the AXIS tool was pre-designated as the definition of a low-quality study. Therefore, a sensitivity analysis was performed to exclude these studies from the meta-analysis to test the robustness of the result.

Only Gong et al met this criterion, and only the analysis for IL-4 permitted exclusion (otherwise there would have been too few studies remaining in the analyses).

Result of meta-analysis: Unchanged finding

Random effects model result: -0.0299

95% CI: (-0.0440; -0.0158)

p-value < 0.0001


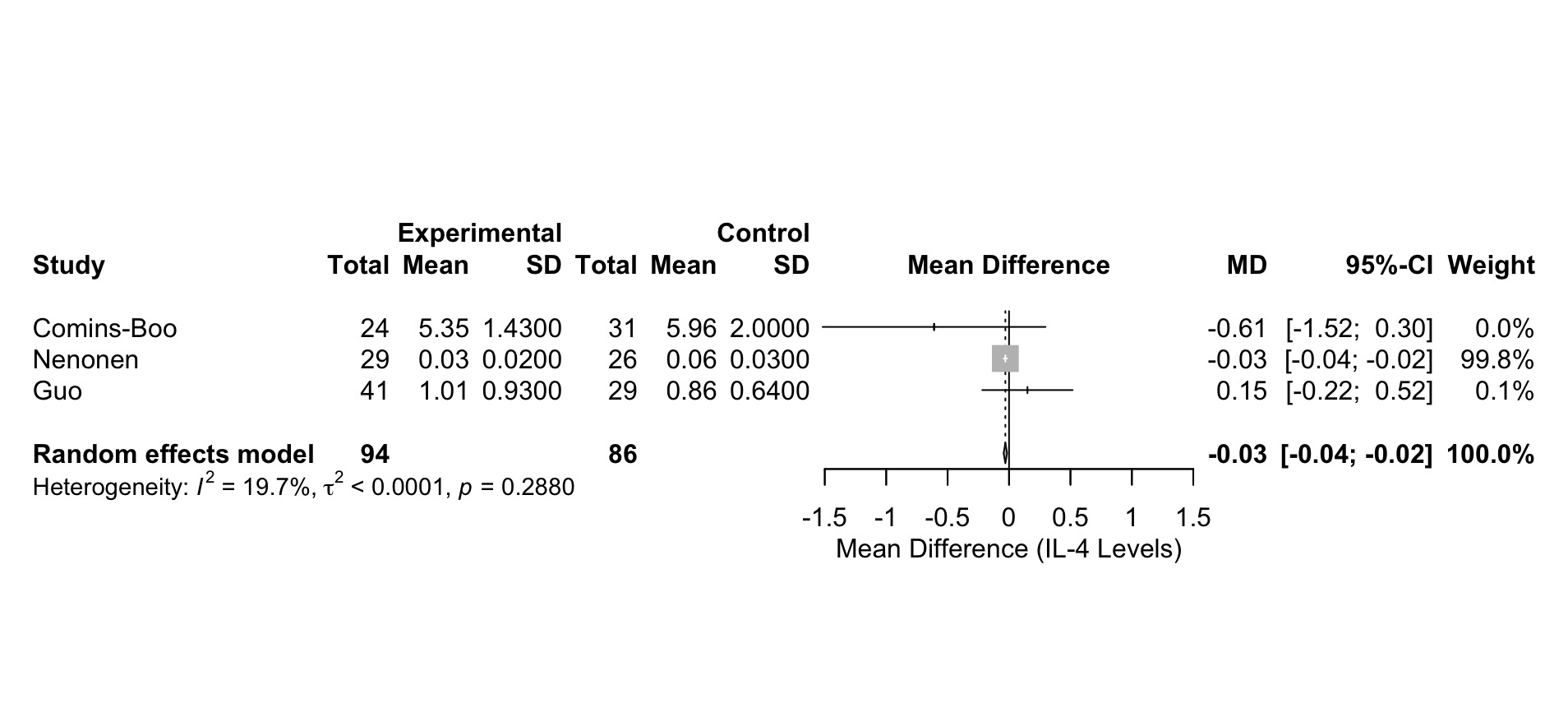

Supplement: Supplementary file 1 [file DataSheet1.docx]
